# P38 mitogen-activated protein kinase promotes dedifferentiation of primary articular chondrocytes in monolayer culture

**DOI:** 10.1111/jcmm.12034

**Published:** 2013-03-11

**Authors:** Derek H Rosenzweig, Sing J Ou, Thomas M Quinn

**Affiliations:** a Soft Tissue Biophysics Laboratory, Department of Chemical Engineering, McGill UniversityMontreal, QC, Canada

**Keywords:** Chondrocyte, MAPK, Signal Transduction, Dedifferentiation, Gene Expression, Extra Cellular Matrix

## Abstract

Articular cartilage is an avascular tissue with poor regenerative capacity following injury, a contributing factor to joint degenerative disease. Cell-based therapies for cartilage tissue regeneration have rapidly advanced; however, expansion of autologous chondrocytes *in vitro* using standard methods causes ‘dedifferentiation’ into fibroblastic cells. Mitogen-activated protein kinase (MAPK) signalling is crucial for chondrocyte metabolism and matrix production, and changes in MAPK signals can affect the phenotype of cultured cells. We investigated the effects of inhibition of MAPK signalling on chondrocyte dedifferentiation during monolayer culture. Blockade of extracellular signal-regulated kinase (ERK) and c-Jun N-terminal kinase (JNK) signalling caused a significant increase in cartilage gene expression, however, also caused up-regulation of fibrotic gene expression. Inhibition of p38 MAPK (p38) caused a significant up-regulation of collagen type II while suppressing collagen type I expression. P38 inhibition also resulted in consistently more organized secretion of collagen type II protein deposits on cell culture surfaces. Follow-on pellet culture of treated cells revealed that MAPK inhibition reduced cell migration from the pellet. ERK and JNK inhibition caused more collagen type I accumulation in pellets *versus* controls while p38 inhibition strongly promoted collagen type II accumulation with no effect on collagen type I. Blockade of all three MAPKs caused increased GAG content in pellets. These results indicate a role for MAPK signalling in chondrocyte phenotype loss during monolayer culture, with a strong contribution from p38 signalling. Thus, blockade of p38 enhances chondrocyte phenotype in monolayer culture and may promote more efficient cartilage tissue regeneration for cell-based therapies.

## Introduction

Articular cartilage comprises a cellular component (chondrocytes) and the extracellular matrix which they produce. This tissue functions to provide load bearing capacity and a continuous and smooth gliding surface for joint locomotion 1. Injured cartilage has poor intrinsic regenerative properties, and trauma can lead to early onset of degenerative joint disease 2–4. At least in the case of post-traumatic cartilage injury, osteoarthritis progression is thought to involve changes in tissue biomechanics, loss in cell viability, sustained inflammatory responses, and changes in chondrocyte biology 2. The final outcome of osteoarthritis is joint deterioration, pain, and disability which generates medical and financial strains world-wide.

Cell-based therapies such as autologous chondrocyte implantation (ACI) are promising clinical techniques which have been used to treat cartilage injuries 5–7. Such methods involve *ex vivo* chondrocyte culture and expansion with minimal extraction of healthy host tissue 8. However, standard monolayer culture techniques 9 and subsequent passaging of expanded primary cell populations often result in phenotype loss termed dedifferentiation 10, 11. Under these conditions, cultured chondrocytes display a dramatic decrease in the chondrogenic marker genes collagen type II, aggrecan and cartilage oligomeric matrix protein (COMP). The dedifferentiated cells start expressing collagen type I and assume a fibroblast-like phenotype which is undesirable for cartilage tissue engineering. Dedifferentiation of chondrocytes resembles at least some of the phenotypic changes observed in osteoarthritis where aggrecan and collagen type II expressions significantly decrease 12.

Rigid culture surfaces 10, enzymatic passaging 13 and high proliferative rates 14 have been proposed as initiating factors in cultured chondrocyte dedifferentiation, and these culture conditions may exert their effects on chondrogenic phenotype through mitogen-activated protein kinase (MAPK) signalling. MAP kinases function within intracellular signalling cascades to relay information relating to external cellular stimuli. These activities can modulate proliferation, differentiation, metabolic processes, apoptosis and other stress responses 15, 16. The MAP kinases consist of extracellular signal-regulated kinase (ERK), p38 MAPK (p38) and c-Jun N-terminal kinase (JNK), all of which are constitutively expressed in most cell types including chondrocytes 15. Recent studies have suggested ERK and p38 are involved in chondrocyte phenotype maintenance 17, 18, however, the exact role of MAP kinases in chondrocyte dedifferentiation remains to be elucidated.

We set out to explore the role of MAP kinase signalling in dedifferentiation of primary chondrocytes in monolayer culture. The commercially available inhibitors PD98059, SB203580 and SP600125 were used to specifically disrupt ERK, p38 and JNK activity respectively. Primary bovine chondrocytes were seeded in monolayer culture in the presence or absence of these inhibitors. Gene expression analyses for the chondrogenic markers collagen type II, aggrecan, COMP and Sox9, as well as the fibrotic marker collagen type I were performed. Follow-on pellet cultures were also performed after treatment with inhibitors, and the efficiency of cartilage-like tissue regeneration was assessed.

## Materials and methods

### Chondrocyte isolation

Primary bovine chondrocytes were isolated as described previously 19, 20. Briefly, knee joints from freshly slaughtered skeletally mature cows were obtained from a local slaughterhouse. Articular cartilage was cut from the femoropatellar groove with a scalpel, and chondrocytes were isolated by enzymatic digestion. Approximately 5 g of tissue was washed in sterile phosphate-buffered saline (PBS) supplemented with antibiotics and cut into 2 mm pieces using a sterile scalpel. The tissue was transferred to a T-75 flask containing 30 ml of chondrocyte growth medium (high-glucose DMEM; 0.1 mM Nonessential Amino Acids; 10 mM HEPES; 1 mM sodium pyruvate; 10% foetal bovine serum; and 1% penicillin-streptomycin-glycine solution) supplemented with 1.5 mg/ml collagenase type II (Invitrogen/Gibco, Burlington, ON, Canada; sterile filtered). Samples were incubated overnight to allow complete digestion of extracellular matrix. The digested mixture was passed through a 100 μm filter (BD Biosciences, Mississauga, ON, Canada) and centrifuged at 200 × *g* for 5 min. The supernatant was removed and pelleted chondrocytes were washed with sterile PBS and centrifuged again at 200 × *g* for 5 min. The supernatant was removed, and cells were resuspended in 10 ml of chondrocyte growth medium. Cells were counted using a hemocytometer. 10,000 cells/cm^2^ were seeded into 6-well plates for experiments.

### Inhibition of MAPK activity

After 5 days in culture, media was removed from chondrocytes (∼70% confluency) and replaced with media containing either 10 μM of the ERK-specific inhibitor PD98059 (referred to as ERKi), the p38-specific inhibitor SB203580 (p38i), the JNK-specific inhibitor SP600125 (JNKi), or the 1% DMSO vehicle (untreated controls) for 2 days (cells reached ∼90% confluency). Cells were then either fixed for immunofluorescence and histological analysis or collected for RNA, protein and redifferentiation assays.

### Chondrocyte redifferentiation

After treatment with MAPK inhibitors or DMSO control, 1 × 10^6^ cells were centrifuged at 500 × *g* for 10 min. in 1.5 ml microfuge tubes to generate a pellet. Chondrocyte growth media was removed and replaced with 500 μl of osteo/chondrogenic differentiation medium (DMEM, 10% FBS, 1.25 mM glutamine, 10 nM dexamethasone, 50 μM/ml ascorbic acid, 1 μM β-glycerophosphate, 5 μg/ml insulin, 0.5 mM 3-isobutyl-1-methylxanthine and 1% penicillin/streptomycin solution) without any further addition of MAPK inhibitors. This culture procedure and medium was modified from previous studies to avoid rapid cell death 21–23. TGFβ was intentionally excluded from this differentiation medium to emphasize effects of MAPK blockade during monolayer culture without the concern of overwhelming growth factor stimulation during follow-on pellet culture. Pellets were incubated in 1.5 ml centrifuge tubes for 6 days until they became firm and then transferred to a 6-well plate (which helped maintain viability) and incubated for an additional 6 days to observe chondrocyte outgrowth. Medium was changed every 2 days. A parallel set of experiments was carried out where pellets remained in the centrifuge tube for 10–12 days, and then fixed with 4% paraformaldehyde and prepared for cryosectioning.

### Reverse transcription and quantitative real-time PCR

RNA was extracted from chondrocytes using TRIzol Reagent (Invitrogen). Following RNA extraction, 500 ng of total RNA was subject to cDNA synthesis using the qScript cDNA synthesis kit following the manufacturer's instructions (Quanta Biosciences, Gaithersburg, MD, USA). Consequently, 1 μl of each cDNA sample was loaded per reaction (in duplicate) using PerfeCTa SYBR Green FastMix (Quanta Biosciences). Standard recommended PCR protocols were performed (50°C for 2 min., 94°C for 10 min., 95°C for 30 sec., 60°C for 1 min., with steps 3 and 4 repeated for 40 cycles) using the ABI 7900 HT Fast Real-Time PCR System (Applied Biosystems, Carlsbad, CA, USA). The average cycle count for each target gene was normalized to GAPDH to give the average delta count (ΔCt) using RQ SDS manager software (Applied Biosystems). Then for each target gene the average ΔCt reading from each experimental cDNA was subtracted from the average ΔCt from the comparative GAPDH endogenous control (ΔΔCt). The average fold change in gene expression of experimental samples compared with controls was calculated by the 2^−ΔΔCt^ method 24. Statistical significance in fold-changes in gene expression was determined using Student's *t*-test (*P* < 0.05). PCR primers for collagen type II, aggrecan, COMP, Sox9, collagen type I and GAPDH were generated exactly as described elsewhere 25.

### Western blotting

Chondrocytes were lysed in lysis buffer [20 mM Tris (pH 7.4), 150 mM NaCl, 1 mM EDTA, 0.5% Triton X-100, 1 mM β-glycerophosphate, supplemented with complete EDTA-free protease inhibitor cocktail]. Twenty microgram of total protein was run on a 10% SDS-PAGE gel and transferred to nitrocellulose membranes. Membranes were blocked in 5% BSA for 30 min. and probed with antibodies against cleaved caspase-3 (1:500), phospho-ERK1/2 (1:2000), phospho-p38 (1:1000) or phospho-JNK (1:1000), MAPKAPK2 (1:500, Bioss Antibodies, Woburn, MA, USA), α-tubulin (1:2000; Abcam, Cambridge, MA, USA), followed by incubation with anti-rabbit horseradish peroxidase-conjugated secondary antibody (1:5000, Cell Signalling, Danvers, MA, USA). Membranes were then washed three times in TBST for 10 min. Western blots were developed using Super Signal West Pico Substrate (Thermo-Fisher, Nepean, ON, Canada) and Kodak BioMax MR film (Perkin Elmer, Woodbridge, ON, Canada).

### Immunofluorescence and histological analysis

For histological analysis, pellet cultures were fixed in 4% paraformaldehyde and embedded in tissue freezing medium (Triangle Biomedical Sciences, Durham, NC, USA). Frozen sections 10 μm thick were cut using a Leica CM3050 S cryomicrotome. Sectioned samples were stained with Safranin O and Alcian Blue for proteoglycan and counterstained with Nuclear Fast Red (Sigma-Aldrich, Oakville, Canada). For immunofluorescence, samples were blocked in permeabilization buffer for 30 min. (PBS, 0.1% Triton X-100 and 1% BSA). Permeabilized samples were then incubated with antibodies against cleaved caspase-3 (1:250, Sigma-Aldrich), collagen type II (1:100, Abcam) or collagen type I (Developmental Hybridoma Bank, University of Iowa) overnight at 4°C. Samples were washed three times in PBS and then incubated with either Alexa Fluor 488 Goat anti-Mouse IgG (1:250, Invitrogen) or TRITC-conjugated Goat anti-Rabbit IgG (1:250, Sigma-Aldrich) for 1 hr at room temperature. Samples were washed and mounted with Fluoroshield with DAPI (Sigma-Aldrich) and visualized on an Olympus IX81 inverted fluorescence microscope. Morphological images were captured using a Zeiss Axiovert 40C microscope equipped with a Canon Powershot A640 digital camera attached to a Zeiss MC80DX 1.0× tube adapter.

### Quantitation of cell proliferation and apoptosis

Isolated chondrocytes were seeded (10,000 cells/cm^2^) on sterilized 22 mm square coverslips (glass) in the presence or absence of MAPK inhibitors. Cells were fixed with 4% paraformaldehyde solution and blocked for 30 min. in permeabilization buffer and probed with antibodies against cleaved caspase-3 and immunofluorescence was performed as above. All images were captured using a 10× objective with MAG Biosystems Software 7.5 (Photometrics, Tucson, AZ, USA). Three random positions per slide were captured from three independent experiments. Positively stained nuclei and peri-nuclear regions were counted and plotted as a percent of total nuclei. Cell growth was determined by trypsinization of cells after 2 days of growth in the presence or absence of MAPK inhibitors, counting cell populations and calculating cell doublings.

## Results

### Chondrocyte dedifferentiation correlates with transient changes in MAPK activity

**Fig. 1:**
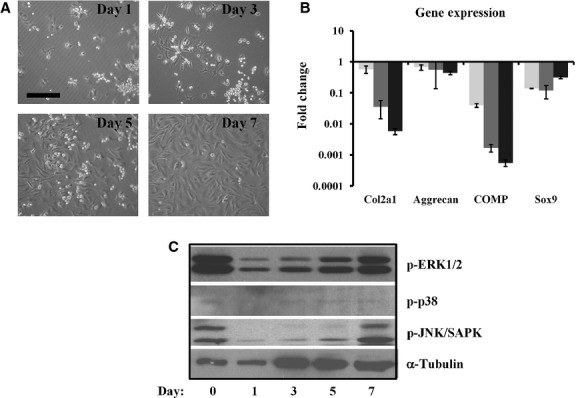
Chondrocytes dedifferentiate in monolayer culture. (**A**) Phase images of primary chondrocytes in culture at indicated times post-seeding. Scale bar: 200 μm. (**B**) qPCR revealed an immediate pattern of dedifferentiation of chondrocytes cultured in monolayer. Light, grey and dark bars represent 3, 5 and 7 days culture, respectively, all normalized to day 1 cultures. Mean ± SEM (*n* = 3). (**C**) Western blot analysis of MAP kinase activity during 7 days of monolayer culture. α-tubulin was used as loading control.

To test for changes in chondrogenic phenotype, 10^5^ primary bovine chondrocytes were seeded on standard polystyrene culture dishes and cultured for 7 days. As expected, cell morphology changed from rounded to fibroblast-like as cells attached and expanded (Fig. 1A). Quantitative real-time PCR analysis revealed significant declines in expression of the chondrogenic genes Col2a1, Aggrecan, COMP and Sox9 after 3, 5 and 7 days of culture, as compared to day 1 controls (Fig. 1B). The fibrotic marker Col1a2 was below detection in all samples tested (data not shown). To assess MAPK activity, Western blot probing for phosphorylated ERK1/2, p38 and JNK/SAPK was performed on cell lysates from freshly isolated chondrocytes (day 0), and cells cultured for 1, 3, 5 and 7 days. Compared with freshly isolated cells, MAPK activity sharply declined upon starting the monolayer culture and then gradually returned to near-control levels over 7 days (Fig. 1C). The gradual increase in MAPK activity from day 1 to 7 correlated with a steady decline in chondrogenic marker expression.

### MAPK inhibition enhances chondrocyte extracellular matrix deposition on culture surfaces

**Fig. 2:**
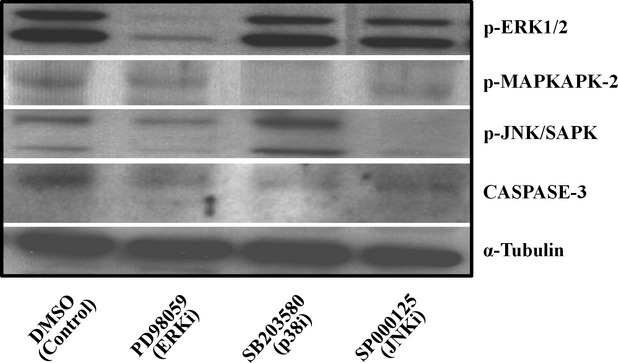
Effects of specific inhibitors on MAPK signalling and apoptosis in cultured chondrocytes. Protein lysates from chondrocytes treated with 10 μm PD98059 (ERKi), SB203580 (p38i), SP600125 (JNKi) or DMSO vehicle (control) were subjected to SDS-PAGE and Western blot and α-tubulin was used as loading control. In all cases, the specific inhibitor was confirmed for blocking its designated kinase. Blockade of JNK caused a slight increase in doublet formation of cleaved caspase-3 (apoptosis marker).

**Fig. 3:**
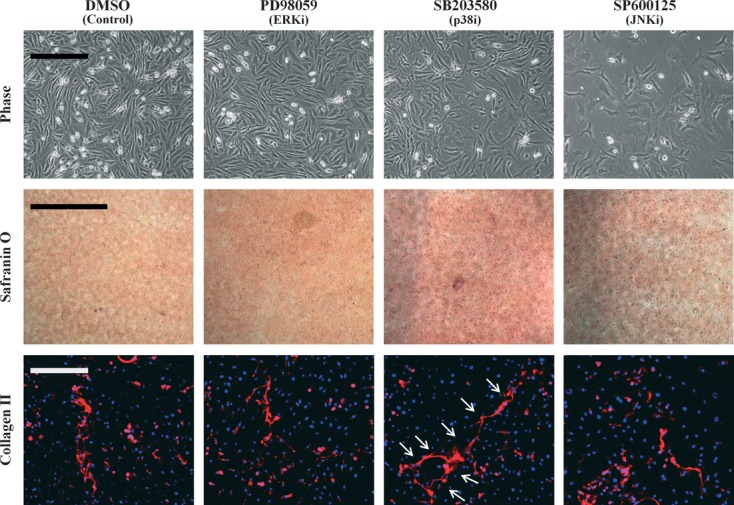
MAPK inhibition affects chondrocyte density and matrix deposition in monolayer culture. Top panels: Phase images of chondrocytes on day 7 after 48 hrs of treatment with specific MAPK inhibitors. Scale bar: 200 μm. Middle panels: Whole-plate Safranin O staining indicating deposited GAG on day 7. Scale bar: 500 μm. Bottom panels: Immunofluorescence microscopy staining for collagen type II deposits on the culture surface on day 7. Arrows indicate more organized networks of collagen type II. Blue: DAPI for nuclei, red: collagen type II. Scale bar: 200 μm.

To ensure suppression of MAPK activity by the specific inhibitors PD98059 (ERKi), SB203580 (p38i) and SP600125 (JNKi), Western blot was performed probing for phospho-ERK1/2, phospho-MAPKAPK2 (a target of active p38) and phospho-JNK/SAPK. In all cases, specific inhibitors were able to selectively abolish MAPK signals (Fig. 2). Caspase-3 activity was also determined in the presence of inhibitors. Chondrocytes treated with JNKi showed an increase in the double band suggesting increased cleaved caspase-3 activity and increased apoptosis. As chondrocyte dedifferentiation was readily evident between 5 and 7 days in monolayer culture (Fig. 1), MAPK inhibitors were added to monolayer cultures between these time-points. Cells were seeded and cultured in growth medium for 5 days, at which point the MAPK inhibitors of ERK, p38 and JNK were introduced to the media, and cultures continued for 2 days. Inhibition of MAPKs did not affect gross morphology, however, JNK inhibition consistently resulted in less cell density on the culture surface (Fig. 3 top panels). In a qualitative comparison with DMSO-treated controls, Safranin O staining of cultures indicated slightly enhanced GAG secretion when MAPKs were inhibited (Fig. 3 middle panels). Inhibition of ERK and JNK resulted in similar deposition of collagen type II as compared with DMSO controls, while inhibition of p38 consistently resulted in longer fibre-like structures and more organized networks of collagen type II (Fig. 3 bottom panels).

### Effects of MAPK inhibition on GAG secretion, cell growth and viability

**Fig. 4:**
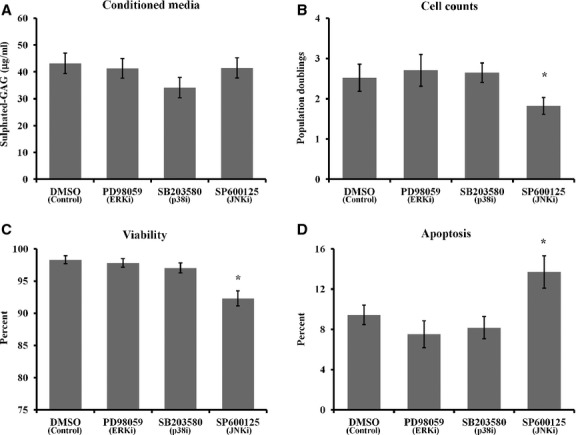
Effects of MAPK inhibition on GAG secretion, proliferation and viability after day 7. (**A**) Conditioned media was collected from treated cultures after day 7 and subjected to DMMB assay to quantify sulfated GAG secretion into the media. (**B**) Population doublings calculated after day 7. (**C**) LIVE/DEAD assay was used to quantify viability. (**D**) Immunofluorescence microscopy was used to visualize cleaved caspase-3, an indicator of apoptosis, and positive cells were quantified. Mean ± SEM (*n* = 3). * indicates *P* < 0.05, Student's *t*-test.

To determine if blockade of MAPK signalling could affect chondrocyte GAG secretion, conditioned media was collected and analysed by DMMB assay to quantify secreted GAG. Inhibition of MAPKs did not alter GAG secretion into the media as compared with DMSO controls (Fig. 4A). Inhibition of JNK caused a significant 30% decrease in population doublings, while ERK and p38 inhibition had no effects (Fig. 4B). LIVE/DEAD assay revealed that inhibition of JNK caused a significant decrease in viability which strongly correlated with a significant increase in cells stained positive for the active apoptosis marker cleaved caspase-3 (Fig. 4C and D). Inhibition of ERK and p38 did not cause changes in viability or apoptosis as compared with DMSO controls.

### MAPK inhibition enhances chondrogenic gene expression in monolayer chondrocyte cultures

**Fig. 5:**
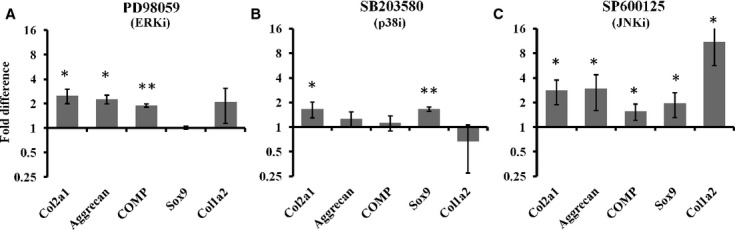
Gene expression analysis. qPCR was performed on cDNA synthesized from isolated RNA of chondrocytes treated with (**A**) ERKi, (**B**) p38i or (**C**) JNKi and compared with DMSO controls. Error bars ± SEM; *n* = 3. * indicates *P* < 0.05, Student's *t*-test.

To assess the effects of MAPK inhibition on chondrocyte phenotype, quantitative real-time PCR (qPCR) was performed. qPCR revealed significantly higher mRNA expression of the chondrogenic markers collagen type II (Col2a1), aggrecan, cartilage oligomeric matrix protein (COMP) and Sox9 in chondrocytes treated with ERK and JNK inhibitors (Fig. 5A and C). However, ERK and JNK blockade also caused up-regulation of the fibrotic marker collagen type I (Col1a2). Inhibition of p38 caused a significant enhancement of collagen type II and Sox9 expression coupled with a strong trend for suppression of collagen type I (Fig. 5B).

### Chondrocyte redifferentiation after MAPK inhibition

**Fig. 6:**
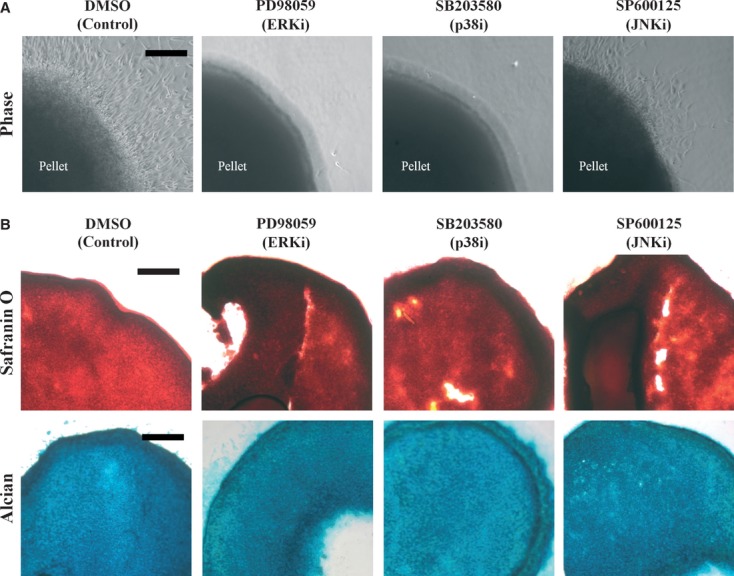
Effects of MAPK inhibition on chondrocyte redifferentiation. Chondrocytes collected after culture in the presence or absence of MAPK inhibitors were subjected to redifferentiation in pellet cultures containing osteo/chondrogenic differentiation medium without further addition of MAPK inhibitors. (**A**) Firm pellets were transferred to 6-well plates after 6 days of culture and an outgrowth assay was performed for another 6 days. Scale bar: 200 μm. (**B**) 10 μm cryosections of pellets were stained with Safranin O and Alcian blue for histological analysis of sulfated GAG content. Scale bar: 200 μm.

Cultured chondrocytes treated with MAPK inhibitors were subjected to micromass pellet cultures to assess their potential to generate *de novo* cartilage-like tissue. In one set of experiments, pellets were transferred from their microcentrifuge tubes after 6 days of culture to 6-well dishes for an additional 6 days, and cell migration from the pellets was qualitatively assessed (Fig. 6A). DMSO control pellets exhibited many cells migrating off the pellet and adhering to the culture surface. Pellets derived from cells treated with ERK and p38 inhibitors displayed no observable cell migration from the pellet. However, pellets derived from cells treated with JNK inhibitor displayed moderate cell migration from the pellet (Fig. 6A).

**Fig. 7:**
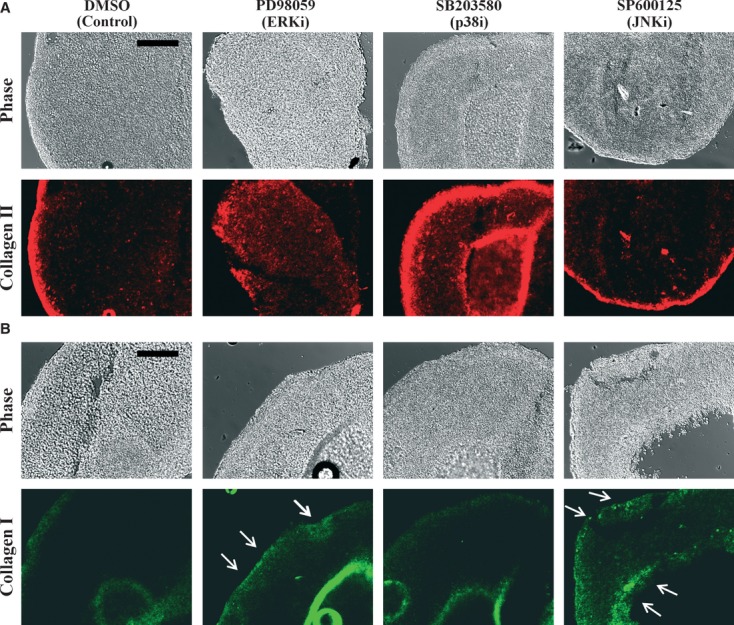
Effects of MAPK inhibition on collagen accumulation in pellet cultures. (**A**) Pellets were stained with antibodies against collagen type II (red) to determine cartilage-like extracellular matrix production. (**B**) Samples were also stained with antibodies against collagen type I (green) to assess fibrosis. Arrows indicate regions of specific collagen type I staining above background intensities. Scale bars: 200 μm.

In a parallel set of experiments, pellets were cultured in microcentrifuge tubes for 12 days and then fixed and prepared for cryosectioning. Inhibition of all three MAPKs resulted in increased Safranin O staining in the pellets compared with DMSO controls; however, no changes in Alcian blue staining were detected (Fig. 6B). Immunofluorescence microscopy revealed a moderate increase in collagen type II in pellets derived from chondrocytes treated with the ERK inhibitor (Fig. 7A). Cells treated with the p38 inhibitor produced pellets containing much more collagen type II compared with both ERK inhibitor and DMSO-treated pellets, while cells treated with JNK inhibitor resulted in pellets with a comparable amount of collagen type II as DMSO controls (Fig. 7A). Immunofluorescence microscopy also revealed collagen type I production in pellets derived from cells treated with ERK and JNK inhibitors (Fig. 7B). Pellets derived from chondrocytes treated with DMSO or the p38 inhibitor produced little to no collagen type I (barely detectable above background; Fig. 7B).

## Discussion

Treatment of primary chondrocyte cultures with specific inhibitors of MAP kinases significantly altered chondrogenic gene expression and changed the efficiency with which cells could produce cartilage-like matrix. Inhibition of ERK or JNK caused up-regulation of chondrogenic gene markers, although also caused an increase in fibrotic gene expression. P38 inhibition in particular caused a significant increase in collagen type II and Sox9 expression while suppressing collagen type I expression, suggesting a blockade of dedifferentiation. PD98059 (ERKi) and SB203580 (p38i) did not cause changes in growth, viability, apoptosis or GAG output of cultured chondrocytes, while SP600125 (JNKi) caused less growth, increased cell death and increased apoptosis. Pellet cultures from cells treated with ERKi had enhanced collagen type II protein expression, increased GAG accumulation, but also displayed increased collagen type I protein expression. Pellets from cells treated with p38i exhibited dramatically increased collagen type II without any detection of collagen type I, suggesting efficient redifferentiation. Blockade of JNK resulted in pellets displaying high levels of collagen type I protein, with collagen type II protein expression not higher than control levels.

As primary chondrocytes adhere and proliferate, they lose their rounded morphology and dedifferentiation begins (Fig. 1). The loss in chondrogenic phenotype is further compounded by passaging with the use of degradative enzymes 10, 11. This dedifferentiation may be inhibited by manipulation of culture conditions including changing the culture surface substrates and reducing passaging during population expansion 19, 20. However, the molecular mechanisms by which these culture modifications exert their positive phenotypic effects are not understood. MAP kinase activity appears to be involved in driving dedifferentiation as it has been established that by the fourth passage, dedifferentiating chondrocytes display increased MAPK activity 17.

In this study, MAPK activity was assessed in primary chondrocytes over the course of 1 week in monolayer culture to gain a better understanding of the signalling pathways associated with dedifferentiation. Changes in MAPK signalling are generally thought to induce negative effects on chondrocyte phenotype 17, 26. Although ERK and p38 signalling have been associated with dedifferentiation in passaged chondrocytes 18, 27, their role in primary cultures have not been thoroughly explored. Present results indicate that ERK, p38 and JNK activities transiently change even in primary (without passaging) chondrocytes (Fig. 1). Intact cartilage tissue exhibits relatively low basal MAPK activity 28. Enzymatic digestion of tissue for primary chondrocyte isolation caused a transient spike in MAPK activity (Fig. 1) which rapidly dropped to a basal level after 1 day in monolayer culture. The MAPK activity then gradually increased from day 1 to 7, correlating with the decrease in cartilage-specific gene expression. These observations suggest a role for MAPK signalling in primary chondrocyte dedifferentiation.

To assess the roles of MAPKs in primary chondrocyte dedifferentiation, pharmacologic inhibitors were administered and phenotype was analysed. Although ERK and JNK inhibition by PD98059 and SP600125 caused increases in chondrogenic gene expression, they also caused increases in fibrotic *Col1a2* expression. The dramatic increase in collagen type I expression observed in SP600125-treated chondrocytes may be attributed to the fact that normal JNK signalling can be anti-fibrotic 29. By inhibiting JNK signalling, cultured chondrocytes lose the ability to prevent fibrosis. Inhibition of p38 activity with SB203580, however, caused an increase in chondrogenic gene expression accompanied by suppression of fibrotic gene expression. Indeed, it has been suggested that p38 signalling is profibrotic 29, and blocking p38 therefore could suppress fibrosis as observed in this study. The effects of p38 inhibition on improved chondrogenic phenotype are in line with recent reports that SB203580 could block the effects of interleukin-1 beta (IL-1β)-induced dedifferentiation 30–32.

There is a distinct similarity between the phenotype of dedifferentiated chondrocytes 10, 11 and chondrocytes isolated from osteoarthritis patients 33. In both cases there is a decrease in sulfated glycosaminoglycan (GAG) content/secretion, decrease in collagen type II and aggrecan expression, and increase in collagen type I expression. We have recently reported that within a cartilage injury model, changes in MAPK activity correlates with changes in chondrocyte viability, gene expression, apoptosis and matrix integrity – phenomenon associated with dedifferentiation and arthritis progression 28. Not surprisingly, changes in MAPK signalling also have importance in arthritis and rheumatism 34. p38 is seemingly the most promising as a therapeutic target against inflammatory arthritis, as inhibition of p38 can block the effects of IL-1 signals (mentioned above) and tumour necrosis factor alpha (TNFα) 35–37. Moreover, p38 activity is much higher in OA chondrocytes, and inhibition of p38 can curb apoptosis and hypertrophic terminal differentiation of cultured OA and healthy chondrocytes 38, 39. Our data suggest that at least in primary non-passaged chondrocytes, p38 signalling also drives the down-regulation of chondrogenic gene expression while increasing fibrotic gene expression, because inhibition of p38 reverses these effects. As IL-1 40 and TNFα have both been shown to be directly involved in chondrocyte dedifferentiation, further studies are required to test if these signals contribute to the present results. In addition to IL-1 and TNFα, the hyaluronic acid receptor CD44 has also been linked to dedifferentiation. Receptor cleavage has been correlated with decreased chondrogenic gene expression and decreased sulfated GAG secretion 41, and probing for CD44 may provide mechanistic insights in future studies.

Micromass 3D pellet cultures are often used to assess the efficiency with which cultured chondrocytes can redifferentiate and produce cartilage-like matrix. Transforming growth factor beta (TGFβ) is often added to chondrogenic medium to enhance redifferentiation and matrix production by chondrocytes 21–23, 42. However, we have previously shown that the redifferentiation capacity of cultured chondrocytes is perhaps better assayed without overwhelming cells with TGFβ signals 19, 20. In this way, variables relating to growth factor dosage are limited and MAPK inhibitor treatment of cells will determine efficiency of tissue regeneration. Although TGFβ classically acts through Smad signalling factors, it has also been shown to activate MAPK signalling 43. Moreover, TGFβ is also able to activate p38 MAPK to drive collagen type I synthesis in mouse glomerular mesangial cells 44. Therefore, avoiding addition of TGFβ to pellet cultures is favourable for interpretation of the effects of MAPK inhibition on chondrocyte phenotype and redifferentiation.

In this study, redifferentiation assays showed that pellets from chondrocytes treated with p38 inhibitors secreted significantly more collagen type II without any detection of collagen type I. These results correlated with recent findings where p38 inhibition strongly blocked the catabolic effects of cytokine stimulation in cartilage explants 37. Specifically, p38 inhibition reduced matrix metalloproteinase and aggrecanase activity, thus elevating collagen type II, aggrecan and GAG content. It is conceivable that inhibition of p38 in our monolayer chondrocyte cultures also reduces catabolic activities; however, further investigation is required for confirmation, Coupled with the gene expression analysis of p38i-treated cells, our data strongly indicate an important role for p38 in dedifferentiation of primary chondrocytes cultured in monolayer. The pellet cultures also indicated that blockade of JNK or ERK signalling caused increased expression of collagen type I, presumably through p38 signals. However, further studies are required to determine upstream and downstream signals controlling these effects.

Mitogen-activated protein kinase activities were assessed in cultured primary chondrocytes to determine their role in dedifferentiation. Inhibition of ERK and JNK signalling increased chondrogenic gene expression, but also increased fibrotic gene expression. Moreover, blockade of JNK signalling reduced population doubling and induced cell death *via* apoptosis. In contrast, inhibition of p38 increased chondrogenic gene expression, suppressed fibrotic gene expression and did not disturb population doublings or viability. Pellet cultures from p38i-treated cells also produced superior cartilage-like tissue compared to other MAPK inhibitors or DMSO controls. These data indicate that under normal monolayer culture conditions, p38 signals act to promote chondrocyte dedifferentiation and inhibition of p38 with SB203580 enhances chondrogenic phenotype, which is desirable for cartilage tissue engineering. On the other hand, inhibition of ERK or JNK promotes fibrosis, rendering these pharmacologic agents undesirable for tissue engineering applications.
